# Prognostic impact of epidermal growth factor receptor (EGFR) expression on loco-regional recurrence after preoperative radiotherapy in rectal cancer

**DOI:** 10.1186/1471-2407-5-62

**Published:** 2005-06-20

**Authors:** David Azria, Frederic Bibeau, Nicolas Barbier, Abderrahim Zouhair, Claire Lemanski, Philippe Rouanet, Marc Ychou, Pierre Senesse, Mahmut Ozsahin, André Pèlegrin, Jean-Bernard Dubois, Simon Thèzenas

**Affiliations:** 1 Department of Radiation Oncology, Val d'Aurelle Cancer Institute, Montpellier, France; 2 INSERM, EMI 0227, Val d'Aurelle Cancer Institute, Montpellier, France; 3 Department of Pathology, Val d'Aurelle Cancer Institute, Montpellier, France; 4 Department of Radiation Oncology, Centre Hospitalier Universitaire Vaudois, Lausanne, Switzerland; 5 Department of Surgical Oncology, Val d'Aurelle Cancer Institute, Montpellier, France; 6 Department of Medical and Digestive Oncology, Val d'Aurelle Cancer Institute, Montpellier, France; 7 Biostatistics Unit, Val d'Aurelle Cancer Institute, Montpellier, France

## Abstract

**Background:**

Epidermal growth factor receptor (EGFR) represents a major target for current radiosensitizing strategies. We wished to ascertain whether a correlation exists between the expression of EGFR and treatment outcome in a group of patients with rectal adenocarcinoma who had undergone preoperative radiotherapy (RT).

**Methods:**

Within a six-year period, 138 patients underwent preoperative radiotherapy and curative surgery for rectal cancer (UICC stages II-III) at our institute. Among them, 77 pretherapeutic tumor biopsies were available for semi-quantitative immunohistochemical investigation evaluating the intensity and the number (extent) of tumor stained cells. Statistical analyses included Cox regression for calculating risk ratios of survival endpoints and logistic regression for determining odds ratios for the development of loco-regional recurrences.

**Results:**

Median age was 64 years (range: 30–88). Initial staging showed 75% and 25% stage II and III tumors, respectively. RT consisted of 44-Gy pelvic irradiation in 2-Gy fractions using 18-MV photons. In 25 very low-rectal-cancer patients the primary tumor received a boost dose of up to 16 Gy for a sphincter-preservation approach. Concomitant chemotherapy was used in 17% of the cases. All patients underwent complete total mesorectal resection. Positive staining (EGFR+) was observed in 43 patients (56%). Median follow-up was 36 months (range: 6–86). Locoregional recurrence rates were 7 and 20% for EGFR extent inferior and superior to 25%, respectively. The corresponding locoregional recurrence-free survival rate at two years was 94% (95% confidence interval, CI, 92–98%) and 84% (CI 95%, 58–95%), respectively (P = 0.06). Multivariate analyses showed a significant correlation between the rate of loco-regional recurrence and three parameters: EGFR extent superior to 25% (hazard ratio = 7.18, CI 95%, 1.17–46, P = 0.037), rectal resection with microscopic residue (hazard ratio = 6.92, CI 95%, 1.18–40.41, P = 0.032), and a total dose of 44 Gy (hazard ratio = 5.78, CI 95%, 1.04–32.05, P = 0.045).

**Conclusion:**

EGFR expression impacts on loco-regional recurrence. Knowledge of expression of EGFR in rectal cancer could contribute to the identification of patients with an increased risk of recurrences, and to the prediction of prognosis.

## Background

In patients with rectal carcinoma, pelvic recurrence is a major source of morbidity and mortality. Despite improvements in surgical approaches, local recurrence may occur in up to 30% of patients treated with surgery including total mesorectal excision [1]. Since 2001, the Dutch Colorectal Cancer Group Trial [2] has confirmed that a short course of radiotherapy (RT) reduced the rate of pelvic recurrence at 3 years, from 10.1% to 3.4%. In addition, a meta-analysis of 19 randomized trials including preoperative RT tends to show that it provides a gain of three percent at 5 years in overall survival [3]. However despite these recent intensive clinical investigations, there is still a need to develop novel strategies in the management of patients with locally advanced rectal cancer.

Advances in the understanding of the molecular biology of rectal cancer have opened many new research directions. Increasing effort has been directed towards developing molecular targeted therapies or searching for molecular markers that are useful either in predicting treatment outcome or in selecting patients for specific molecular targeted therapies, based on particular tumor characteristics. None of the recent studies has identified convincing data to warrant routine clinical application of any marker such as p53 [4,5], or apoptosis regulators [6].

To date, no data have become available that shed light on the impact of EGFR expression on local and distant relapse in patients treated with preoperative RT and extensive local surgery i.e. abdominoperineal excision or low anterior resection with total mesorectal excision. We present here the prognostic impact of EGFR expression on locoregional recurrence in 77 patients treated with preoperative RT at our institute.

## Methods

### Patient selection and pretreatment evaluation

Within a six-year period (April 1996 and September 2002), 138 patients underwent preoperative radiotherapy and curative surgery for rectal cancer (UICC stages II-III) at the Val d'Aurelle Cancer Institute of Montpellier, France. A carcinoma was considered a primary rectal carcinoma if it was located in the lower third (<6 cm from the anal verge), middle third (6–12 cm), and upper third of the rectum (above 12 cm). Pretherapy biopsies were available for analysis in 77 patients and were evaluable for the statistical results. Diagnostic and distant disease extension studies consisted of colorectal endoscopy with biopsies, rectal ultrasonography (uT), presurgical carcinoembryonic antigen (CEA) value, abdominal and pelvic computed tomography (CT) scans, chest X-ray or CT-scan and routine laboratory studies. All patients were metastasis-free at diagnosis.

### EGFR immunohistochemical assay (IHC)

**Figure 1:**
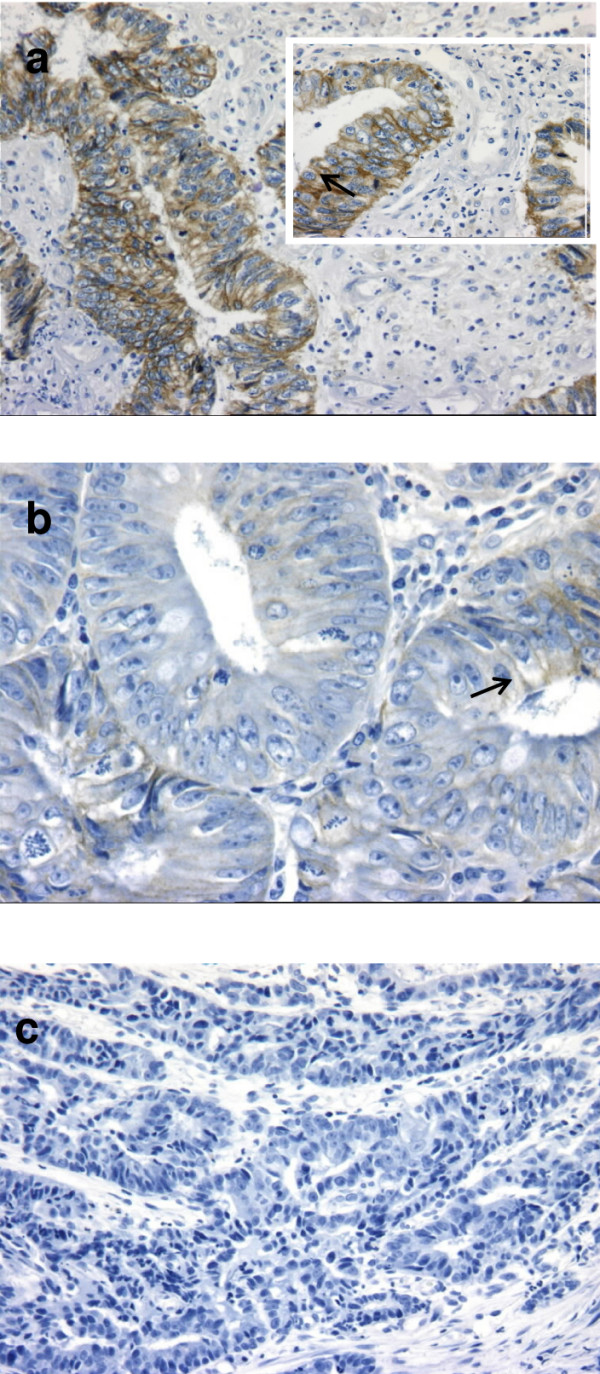
**Patterns of EGFR expression. **a) Case with an extent positivity ≥25% with strong membrane staining of tumor cells. Insert and arrow: note the membranous positivity pattern (immunoperoxydase × 100 and × 400). b) Case with an extent positivity <25% with weak membrane staining of some tumor cells (arrow) (immunoperoxydase × 400). c) Case with negative staining (immunoperoxydase × 200).

IHC of the tumor biopsies was performed by using the Dako autostainer (DakoCytomation, Glostrup, Denmark) and the EGFR Pharm Dx kit^® ^K 1494 (Dako Cytomation, Glostrup, Denmark), according to the manufacturer's instructions with the reagents supplied with the kit. Briefly, sections of 3 μm were mounted on silanized slides and allowed to dry overnight at 37°C. After deparaffinization and rehydratation, slides were incubated with 3% hydrogen peroxide solution for 5 min. After a washing procedure with the supplied buffer, tissue sections were covered for 5 min with protein K solution. The slides were then incubated for 30 min with the primary mouse anti-EGFR MAb (clone 2-18C9), which binds to a formalin-resistant epitope near the ligand-binding site on the extra cellular domain of the EGFR and recognizes both wild type and mutant type (vIII). After two rinses in buffer, the slides were incubated with the detection system for 30 min (labeled polymer-HRP). Tissue staining was visualized with a DAB substrate chromogen solution. Slides were counterstained with hematoxylin, dehydrated, and mounted. Negative control sections were processed without the primary antibody but with an irrelevant murine IgG1 supplied with the kit. Negative and positive control cell slides provided with the kit EGFR Pharm Dx^® ^were also used, to ensure that each assay run was performed appropriately and according to protocol specifications. Furthermore, perineurium was considered as a positive internal control on tumor slides. EGFR assessment was realized according to the EGFR Pharm Dx^® ^scoring guidelines. Results were reported as positive when a complete or incomplete circumferential membrane staining was observed in at least 1% of the tumor cells. Staining was defined as any IHC staining of tumor cell membranes above background level, i.e., weak, moderate, or strong. Absence of or cytoplasm staining was reported as negative. In addition to these standardized criteria, the pathologist performed a semi-quantitative evaluation reporting both intensity and percentage (extent) of tumor cells staining blinded to clinical data (Figure 1).

### Preoperative radiation therapy (RT) and surgical modalities

Patients were treated in supine position with a 3-field (posterior and two opposed laterals) isocentric technique using 18-MV photon beams daily, five times a week. The daily dose at the isocenter was 2 Gy; the total dose to the entire pelvis was 44 Gy. In 25 very low-rectal-cancer patients, primary tumor received a boost dose of up to 16 Gy for a sphincter-preservation approach. Clinical target volume (CTV) included the tumor and the entire rectum, the anterior wall of the sacrum and the posterior wall of the prostate or vagina, and the following lymph nodes: perirectal, presacral, hypogastric, obturator, and low common iliac nodes. The planning target volume (PTV) included the clinical target volume plus a 1–1.5-cm margin. The superior margin was the L5-S1 interspace in most patients; in some patients with tumors very close to the anus, however, the cranial margin was placed somewhat lower, but always at least 5 cm above the tumor area. The lateral margins were 1 cm outside the bony margins of the true pelvis. The posterior margin was placed just posterior to the sacrum. The anterior margin was dependent on the anterior extension (gross tumor volume, GTV) of the primary tumor. Individually shaped blocks were used to shield normal tissues. The boost volume covered the primary tumor plus a 1.5-cm margin using a 3-field (posterior and oblique) technique. Standard or CT-scan simulation was used. With the CT-scan simulator (Picker PQ 2000 + ACQSIM), GTV, CTV, and PTV were determined as defined above, the treated volume and the irradiated volume according to the ICRU report 50 [7]. Fields were marked during initial CT-scan simulation after the ICRU reference was calculated.

Thirteen patients (17%) received concomitant chemotherapy. In eight patients, the chemotherapy regimen consisted of continuous infusion of 200–250 mg/m^2^/day of 5-fluorouracil (5-FU) alone beginning on the first day of radiation therapy, five days a week for 5 weeks. Oxaliplatin (40 mg/m^2^/day at days 1, 8, 15, 22, and 29) and leucovorin (100 mg/m^2^/day at days 1–2, 15–16, and 29–30) were added at the same protocol of 5-FU for two and three patients, respectively.

Median time between the last day of radiotherapy and surgery was 41 days (range: 13–97). The choice of the surgical procedure was at the surgeon's discretion. In all cases, the entire mesorectum was removed. Specimens were inked for lateral margin determination. R1 resection was defined as lateral clearance less than one mm.

Clinical, operative, and histopathological data were recorded prospectively in a computerized registry database including patient age, gender, tumor site, tumor stage according to UICC stage [8], histological differentiation, gross morphology, tumor size, local invasion, nodal status and type of surgery.

### Follow-up

All patients were seen on regular follow-up including clinical history, physical examination, laboratory investigations, abdominal ultrasonography, chest X-ray, and endoscopy (sigmoidoscopy after 6 months, total colonoscopy after one year). They were followed semi-annually during the period of 2–5 years postoperatively until death or the closing date of the study (July 2004). Any regrowth of tumor within the pelvis was considered as a local recurrence. The diagnosis of a pelvic recurrence was preferably proven by histology and/or cytology; however, in the majority of cases, the diagnosis was made on clinical or radiological grounds. Data collected were entered prospectively into the registry database. Median follow-up of all patients was 36 months (range: 6–86 months).

### Statistical methods

The characteristics of EGFR staining were examined for correlation with tumor- and patient-related prognostic factors. The cut-off of 25% of EGFR staining corresponded to the third quartile of EGFR extent and was then selected for all statistical correlations. Categorical variables were reported by means of contingency tables. Furthermore, for continuous variables the median and range were computed.

To investigate the association between trial features, univariate statistical analyses were performed using Pearson's Chi-2 test or Fisher's exact test when applicable.

Survival times to all events were measured from the day of surgery to the time of the event or to last news if no event occurred. Relapse-free survival (RFS; event was all relapse), locoregional recurrence-free survival (LRFS; event was locoregional recurrence), and distant metastasis-free survival (MFS; event was distant metastasis relapse) rates were estimated according to the Kaplan-Meier method. Patients not presenting the event of interest were considered censored at the last known follow up of time. Survival curves were drawn, and the logrank test was performed to assess differences between the groups.

Cox's proportional hazards regression using a stepwise selection procedure was used to investigate prognostic factors. Hazards ratios with 95% confidence interval, CI, are presented.

All P values reported were two-sided, and differences were considered as significant at the 5% level. Data were analyzed with software STATA 7.0 (Stata Corporation, College Station, TX, USA).

## Results

### EGFR expression

The semi-quantitative analysis of EGFR expression is summarized in Table 1. Fifty-six percent of the cases demonstrated EGFR expression, and 44% had negative staining. EGFR staining extent superior to 25% was observed in 26% of the cases, and the staining intensity was graded as strong in 8%. Strong staining intensity occurred statistically more frequently in those cases with EGFR extent ≥25% than in those with <25% (P = 0.018).

### EGFR and clinical characteristics of the study population

A total of 77 patients were evaluable for EGFR expression. Median age was 64 years (range: 30–88). Twenty-six (34%) were female and 19 (25%) were staged as stage III patients. A majority of patients presented T3/T4 (72%) rectal tumor. Initial tumor was located in the lower third (n = 52, 68%), middle third (n = 17, 22%), and upper third (n = 8, 10%) of the rectum. Twenty-five patients (32%) received a total radiation dose of 60 Gy for a sphincter-conserving approach. Thirteen patients (17%) and 25 patients (32%) received preoperative concomitant chemo-radiotherapy and adjuvant chemotherapy, respectively. Microscopic incomplete surgery (R1) was achieved in 7 patients (9%), and all corresponded to initially T4 tumors.

We compared the distribution of patients and tumor characteristics and treatment according to EGFR expression (staining intensity and extent) to assess the presence of potential imbalances in the known prognostic variables. Table 2 shows no significant differences between the groups in the distribution of known clinical prognostic indicators of loco-regional control and survival, *i.e*., age, gender, stage group, tumor location, preoperative total dose RT, concomitant chemo-radiotherapy, type of surgery, resection margins. Neither was any imbalance observed for patients who received adjuvant chemotherapy.

### EGFR expression and relapse

Overall tumor progression, caused by local recurrence alone (n = 1, 1.3%), distant metastases alone (n = 8, 10.4%), and both of them (n = 7, 9.1%) occurred in 16 patients (20.8%).

**Figure 2:**
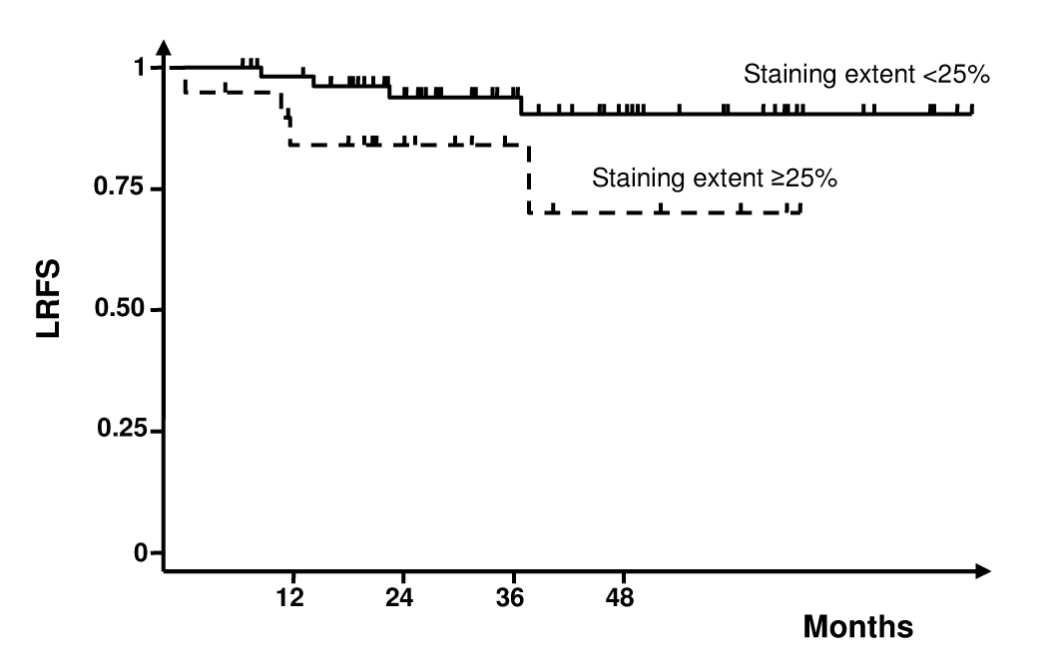
Locoregional recurrence-free survival curves according to epidermal growth factor receptor (EGFR) expression extent.

Patients with EGFR extent ≥25% had a higher locoregional recurrence rate (20% vs 7%). The two-year LRFS rate was 94% (92–98%) in patients with EGFR extent <25% and 84% (58–95%) in patients with EGFR extent ≥25% with a borderline statistical difference (P = 0.06, Figure 2).

An EGFR extent of ≥25% was associated with poorer MFS (84% [59–95%] vs 95% [84–98%]) but the difference did not achieve statistical significance. Metastatic evolution corresponded to lung, liver, peritoneum, bone, and brain in 60%, 40%, 26%, 13% and 7%, respectively. No difference was observed in the pattern of metastatic failure according to EGFR status.

Patients with strong EGFR staining intensity had a higher loco-regional recurrence rate (17% vs 10%) and a poorer RFS than those with negative to moderate staining intensity but without significant statistical difference.

Univariate analysis did not show any significant association of tumor local recurrence with age (P = 0.48), gender (P = 0.81), UICC stage III (P = 0.08), tumor location (P = 0.60), preoperative chemotherapy (P = 0.73), preoperative RT (P = 0.26), type of surgery (P = 0.66), resection margins (P = 0.10), and adjuvant chemotherapy (P = 0.75). To adjust for prognostic factors, the clinical parameters described in Table 2 were included in the multivariate analysis using the Cox proportional hazards model, i.e., EGFR extent (<25% vs ≥25%), resection (complete [R0] vs R1), tumor stage (II vs III), preoperative total dose RT (44 vs 60 Gy), gender, tumor location (lower third vs other thirds), type of surgery, resection margins, age (≤64 vs >64 years old), preoperative concomitant chemo-radiotherapy (no vs yes), EGFR intensity (negative to moderate vs strong), delay from the last day of RT to the day of surgery (≤41 vs >41 days) and uT.

EGFR extent expression, R1 resection, and 44-Gy total dose radiation were the independent prognostic factors that predicted locoregional failure with P values of 0.037, 0.032, and 0.045, respectively (Table 3). For both RFS and MFS, stage III tumor was detected as an independent prognostic factor with P values of 0.024 (hazard ratio = 4.08, CI 95%, 1.21–13.82) and 0.023 (hazard ratio = 4, CI 95%, 1.22–13.13), respectively. Concomitant preoperative chemo-radiotherapy was detected as a potential prognostic factor for RFS and MFS but statistical analysis showed only a trend towards significance P = 0.057 and 0.070, respectively. Margin resection <1 mm was also detected as a significant prognostic factor for MFS (hazard ratio = 5.03, CI 95%, 1.02–24.78, P = 0.047). EGFR expression predicted neither RFS (hazard ratio = 1.11, CI 95%, 0.28–4.46, P = 0.88) nor OS (hazard ratio = 1.26, CI 95%, 0.30–5.41, P = 0.753).

## Discussion

The identification of parameters that reflect biological behavior of individual cancer tissues correlating with tumor aggressiveness is a key determinant of prognosis and a fundamental issue for the improvement of cancer therapy. Despite recent progress in defining the molecular mechanisms of cancer development and tumor progression, only a few individual biomarkers providing prognostic information have been identified. Among them, the EGFR pathways attracted the most attention of cancer investigators.

EGFR (HER1), a transmembrane glycoprotein, is a member of the large receptor tyrosine family encoded by a gene located in human chromosome 7p12. EGFR exists in inactive monomer form or in active dimer form. Dimerization can take place between different receptors in order to develop homologue (homodimers) or heterologue (heterodimers) dimers [9]. In either normal or malignant cells, the activation of EGF receptor cascades may have multiple consequences such as cell growth, differentiation, and proliferation. EGF receptor cascades may also promote malignant transformation, angiogenesis, and/or metastatic dissemination [10,11].

In addition, the cell membrane has been known for some time to be a secondary target for ionizing radiation. This phenomenon may provoke the pathways of mitogen-activated protein kinase (MAPK), phosphatidyl inositol-3-phosphate kinase (PI3K), and MAPK8 activation [12], which can modulate cell proliferation or death. Preclinical and clinical studies associate EGFR expression with radioresistance [13-16]. Ionizing radiation produces several types of cellular response via activation of multiple transduction pathways resulting in cell death, differentiation, or proliferation. Following irradiation, the MAPK pathway was recently reported to be a cellular "SOS" signal initiator starting from EGF receptors [17]. MAPK pathway activation via EGFR receptors was reported in many malignant human cell lines [17-19]. This activation is similar to the one produced by physiological concentrations of EGF (0.1 nM), and seems to act as a radioprotector [16,17,19,20]. Moreover, it has been recently shown that EGF-receptor and MAPK signal pathway activation following ionizing radiation depends on the proteolytic clivage of TGFβ precursor and functional activation of autocrine TGFβ [21]. STAT-3 signal pathway activation by phosphorylation via EGF receptors can be initiated by ionizing radiation, and it results with a radioprotective effect by apoptosis inhibition [22-24]. An inverse relation between the number of EGF receptors and tumor radiocurability is reported in several murine cell lines. In these models, radiation-induced apoptosis was decreased when important levels of EGF receptor were expressed on the cells [25,26]. Clinical consequences of these findings would be tailoring treatment according to a simple predictive assay of radiosensitivity based on the EGF-receptor expression. Clinical data pertaining to the relationship between EGFR expression and the success of radiotherapy are sparse and equivocal. Nevertheless, with respect to squamous-cell cancer of the head and neck, EGFR is among the best-studied examples [27-33], and positive and negative correlations between EGFR levels and tumor recurrences were reported in laryngeal cancers after radiotherapy [34-36]. The relationship of EGFR levels to the prognosis in unresectable pharyngeal or nasopharyngeal cancer patients treated by chemo-radiotherapy was recently reported [37-39].

In colorectal cancer, EGFR expression was evaluated in resected tumors [40]. The authors found significantly higher EGFR levels in stage III cancers than in stages I and II. It was then concluded that high EGFR expression is associated with poor prognosis. Another group [41], found 72 cases of EGFR-positive expression in 82 resected colorectal adenocarcinomas (88%). The extent of EGFR expression (>50%) revealed significant differences in survival times. In our study, a significant correlation between the positive tumor cell percentage greater than 25% and the rate of locoregional recurrence was detected (P = 0.037).

We did not assess the predictive value of EGFR on tumor response after preoperative treatment. This question was recently tackled by Giralt et al [42]. The authors analyzed EGFR expression of 45 locally advanced-rectal-cancer patients treated with preoperative radiotherapy and total mesorectal resection. Immunochemistry for EGFR was determined at the preradiation diagnostic biopsy and in the resected specimens. EGFR positivity was observed in 29 of 45 tumors (64%) and was associated with neither clinical tumor stage nor clinical nodal stage. The overall response rate was 34% in EGFR positive patients vs. 62% in those who did not express EGFR (P = 0.07). Only one of the seven pathologic complete remission patients was EGFR positive (P = 0.003). The link between the positive EGFR expression and the microscopic response on surgical specimen seems to be logical, but we fail to assess it in our series. Such a relationship should be based on a large tumor sampling and needs a very strict procedure at the macroscopic level, to ensure that the whole tumor is analyzed after a neoadjuvant treatment. An exhaustive tissue material should allow a precise analysis of the entire spectrum of tumor regression, i.e., complete, partial or none, as it has been proposed by Dvorack et al [43]. It should then be of interest to correlate these well documented histopathological data with biological parameters such as EGFR.

In our study EGFR expression was not found to be an independent prognostic factor for overall survival in patients with rectal cancer. Other studies, described above [40,41], have reported variable results making it difficult to draw firm conclusions about a possible relationship between EGFR expression and overall survival. Probably, the variation in results is due to (i) the use of different laboratory tests, (ii) varying extent of follow-up, and (iii) heterogeneity in the population of colon- and rectum-cancer patients. (i) EGFR assessment in previous studies was obtained by using different antibodies, different methods of antigen retrieval, and different cut-off values. In this study, we used uniform reagents provided by a kit allowing minimized variations in results and a reproducible method. Therefore, our results detected a low percentage of EGFR immunopositivity (56%) as compared with other colorectal cancer trials [44] probably due to the numerous IHC techniques. (ii) In this study we did not have a sufficiently long follow-up to give definitive conclusions on the prognostic impact on EGFR expression and overall survival. In fact, our analysis was only based on a group of patients with rectal carcinoma, a disease with a natural history different from that of colon carcinoma especially with respect to the tendency to recur locally. (iii) Reasons for recurrence after curative resection for colorectal carcinoma are not completely clear. Several theories have been put forward including, amongst others, microscopic deposits in the lymphatics, inadequate distant and lateral resection margins, exfoliated tumor cells at time of surgery, presence of malignant cells in the anastomosis and, finally, tumor aggressiveness related to biological behaviour. It is known that reported recurrence after resection for rectal carcinoma is commonly higher than after colon carcinoma [45,46], and differences in prognosis have also been reported between high and low rectal carcinomas [47]. Risk factors that have previously been associated with increased recurrence rates include amongst others patient age, gender, tumor stage, site of lesion (colon vs rectum), infiltration of adjacent organs, histopathological criteria, tumor size, lymph-node involvement and radial resection margins [45,46,48-50]. In rectal cancer, in particular, the impact of surgery and adequate lymph-node dissection related to the risk of local recurrence have been highlighted [51]. Several studies have evaluated the prognostic significance of EGFR on survival in colorectal cancer but, to our knowledge, not specifically focusing on rectal cancer recurrence.

In our study, multivariate statistical model identified EGFR expression as a significant independent predictor of recurrence following preoperative and curative surgery for rectal cancer. Two possible explanations for this relationship are to be considered. Firstly, EGFR overexpressing tumors exhibit a more aggressive behavior leading to more pelvic recurrences and in a lesser extent to more distant metastases. A second possible explanation is that EGFR overexpressing tumors present decreased intrinsic radiosensitivity as explained at the first part of this discussion and lead to more pelvic recurrences. This explanation is supported by the fact that the majority of the observed recurrences in our study appeared in the irradiated areas.

Therapeutic approaches targeting EGFR signaling pathways either alone or in combination with radiation or cytotoxic agents are being intensively investigated [52]. Strategies that are in various stages of development include blockade of the extracellular receptor domain [44] and inhibition of the intracellular tyrosine kinase activity [53]. Data presented in Figure 2 suggest that tumor radiation-sensitization through the inhibition of EGFR signaling could yield a therapeutic gain by increasing the locoregional control rate in patients with EGFR-overexpressing rectal cancer.

## Conclusion

Knowledge of expression of EGFR in rectal cancer can contribute to the identification of patients with an increased risk of recurrences. Our results have to be related to several confounding factors such as the small number of events and the retrospective approach. Nevertheless, the potential of introducing routine EGFR immunohistochemistry as a diagnostic tool into the clinical practice of rectal cancer management still has to be undertaken, and may allow clinicians to deliver targeted therapies even in patients with a poorer prognosis.

## Competing interests

The author(s) declare that they have no competing interests.

## Authors' contributions

DA conceived the study, participated in its design and coordination. FB performed all EGFR immunohistochemical analyses. NB made acquisition of the data. AZ, MO, MY, CL, PR, AP, and JBD participated in the design of the study, in its analysis and in the interpretation of the data. DA, FB, ST, and MO drafted the manuscript. ST performed all statistical analyses. All authors read and approved the final manuscript.

## Pre-publication history

The pre-publication history for this paper can be accessed here:



## Figures and Tables

**Table 1:** EGFR immunohistochemical staining characteristics in rectal-cancer patients

EGFR staining	Patients^a ^(%)	EGFR extent	Patients^a ^(%)	EGFR intensity	Patients^a ^(%)
Negative	34 (44)	-	-	-	-
Positive	43 (56)	<25%	23 (30)	Weak and moderate	21 (27)
				Strong	2 (3)
		≥25%	20 (26)	Weak and moderate	16 (21)
				Strong	4 (5)

^a^Number of patients

**Table 2:** EGFR expression and clinical characteristics of the study population

	EGFR extent		P value	EGFR intensity		P value
				
Parameters	<25%	≥25%		Negative to moderate	Strong	
Age (y)						
≤64	26 (45.6)^a^	13 (65)		36 (50.7)	3 (50)	
>64	31 (54.4)	7 (35)	0.14	35 (49.3)	3 (50)	0.97
Gender						
Male	38 (66.7)	13 (65)		47 (66.2)	4 (66.7)	
Female	19 (33.3)	7 (35)	0.89	24 (33.8)	2 (33.3)	0.98
Stage group^b^						
II	43 (75.4)	15 (75)		54 (76.1)	4 (66.7)	
III	14 (24.6)	5 (25)	0.97	17 (23.9)	2 (33.3)	0.61
Tumor location						
Lower third	39 (68.4)	13 (65)		46 (64.8)	6 (100)	
Middle third	12 (21.1)	5 (25)		17 (23.9)	-	
Upper third	6 (10.5)	2 (10)	0.96	8 (11.3)	-	0.21
Preoperative						
RT-CT^c^	8 (14)	5 (25)	0.26	11 (15.5)	2 (33.3)	0.26
Preoperative RT						
44 Gy	40 (70.2)	12 (60)		48 (67.6)	4 (66.7)	
60 Gy	17 (28.8)	8 (40)	0.40	23 (32.4)	2 (33.3)	0.96
Type of surgery^d^						
AP	46 (80.7)	17 (85)		58 (81.7)	5 (83.3)	
LAR	11 (19.3)	3 (15)	0.67	13 (18.3)	1 (16.7)	0.92
Resection						
Margins						
-	52 (93)	17 (85)		63 (90)	6 (100)	
+	4 (7)	3 (15)	0.30	7 (10)	-	0.41
Adjuvant						
chemotherapy	16 (28.1)	9 (45)	0.16	24 (33.8)	1 (16.7)	0.39

^a^Data are presented as number of patients, with the percentage in parentheses.

^b^According to AJCC (American Joint Committee on Cancer) 1997.

^c^Preoperative chemo-radiotherapy.

^d^AP, abdominoperineal excision; LAR, low anterior resection

**Table 3:** Cox multivariate regression analysis for loco-regional recurrence-free survival (LRFS)^a^

Prognostic variable		Hazard ratio	95% CI^b^	Pvalue
EGFR extent	<25% ≥25%	1 7.18	1.12 to 46	0.037
Resection	R0 R1	1 6.92	1.18 to 40.42	0.032
Preoperative RT	60 Gy 44 Gy	1 5.78	1.04 to 32.06	0.045

^a^Results were adjusted on tumor stage, gender, type of surgery, surgical margin, age, preoperative radio-chemotherapy, EGFR intensity, delay from radiotherapy to surgery, and uT

^b^CI, confidence interval
